# Supplementary material for: Emergence of trait variability through the lens of nitrogen assimilation in Prochlorococcus
Source: eLife. 2019 Feb 1;8:e41043. doi: 10.7554/eLife.41043 (PMC6370341; doi:10.7554/eLife.41043)
Supplement: Transparent reporting form [file elife-41043-transrepform.docx]

***eLife’s* transparent reporting form**

We encourage authors to provide detailed information *within their submission* to facilitate the interpretation and replication of experiments. Authors can upload supporting documentation to indicate the use of appropriate reporting guidelines for health-related research (see [EQUATOR Network](http://www.equator-network.org/%20)), life science research (see the [BioSharing Information Resource](https://biosharing.org/)), or the [ARRIVE guidelines](http://www.plosbiology.org/article/info:doi/10.1371/journal.pbio.1000412) for reporting work involving animal research. Where applicable, authors should refer to any relevant reporting standards documents in this form.

If you have any questions, please consult our Journal Policies and/or contact us: [editorial@elifesciences.org](mailto:editorial@elifesciences.org).

**Sample-size estimation**

- You should state whether an appropriate sample size was computed when the study was being designed
- You should state the statistical method of sample size computation and any required assumptions
- If no explicit power analysis was used, you should describe how you decided what sample (replicate) size (number) to use

Please outline where this information can be found within the submission (e.g., sections or figure legends), or explain why this information doesn’t apply to your submission:

No explicit power analysis was used to predetermine sample size. Sample size was limited by sequencing costs and we used all *Prochlorococcus* and marine *Synechococcus* (subcluster 5.1) genomes that were available in Integrated Microbial Genomes <https://img.jgi.doe.gov/cgi-bin/m/main.cgi> (IMG).

The number of genomes used in the final data set, after exclusion criteria were applied, are detailed in the Methods and in Supplementary file 1.

**Replicates**

- You should report how often each experiment was performed
- You should include a definition of biological versus technical replication
- The data obtained should be provided and sufficient information should be provided to indicate the number of independent biological and/or technical replicates
- If you encountered any outliers, you should describe how these were handled
- Criteria for exclusion/inclusion of data should be clearly stated
- High-throughput sequence data should be uploaded before submission, with a private link for reviewers provided (these are available from both GEO and ArrayExpress)

Please outline where this information can be found within the submission (e.g., sections or figure legends), or explain why this information doesn’t apply to your submission:

We arrived at our main data set using the exclusion criteria detailed in the “Dataset” subsection of the Methods. Briefly, we excluded single cell genomes with very low genome recovery and single cell genome assemblies that contained contigs from other organisms.

Further exclusion criteria for individual analytical pipelines are documented in the following subsections of the Methods: “Analysis of covariation in gene content”; “Genome location and synteny”; “Genetic distance of representative genes”; and “Beta diversity analysis.”

Replication of the beta diversity analysis is detailed in the Methods. We consider these technical replicates given that the single cells used in each replicate analysis were from the same discrete population. Here we chose two discrete populations to compare (ones with the highest number of single cell genomes). 9 genomes from each population were subsampled 3 times and used for beta diversity analyses. The results of each subsampled analysis run are presented in Table 2.

The genomic data used in our study is detailed in the following paper:

Berube, P. M., S.J. Biller, T. Hackl, S.L. Hogle, B.M. Satinsky, J.W. Becker, R. Braakman, S.B. Collins, L. Kelly, J. Berta-Thompson, A. Coe, K. Bergauer, H.A. Bouman, T.J. Browning, D. De Corte, C. Hassler, Y. Hulata, J.E. Jacquot, E.W. Maas, T. Reinthaler, E. Sintes, T. Yokokawa, D. Lindell, R. Stepanauskas, and S.W. Chisholm. 2018. Single cell genomes of *Prochlorococcus*, *Synechococcus*, and sympatric microbes from diverse marine environments. Sci Data **In Press**.

Additionally, we produced closed genome sequences for 4 Prochlorococcus strains. These genomes have been deposited with IMG and have the following accession numbers: 2681812899, 2681812900, 2681812901, 2681812859.

**Statistical reporting**

- Statistical analysis methods should be described and justified
- Raw data should be presented in figures whenever informative to do so (typically when N per group is less than 10)
- For each experiment, you should identify the statistical tests used, exact values of N, definitions of center, methods of multiple test correction, and dispersion and precision measures (e.g., mean, median, SD, SEM, confidence intervals; and, for the major substantive results, a measure of effect size (e.g., Pearson's r, Cohen's d)
- Report exact p-values wherever possible alongside the summary statistics and 95% confidence intervals. These should be reported for all key questions and not only when the p-value is less than 0.05.

Please outline where this information can be found within the submission (e.g., sections or figure legends), or explain why this information doesn’t apply to your submission:

The statistical test and method of multiple hypothesis correction used for the gene enrichment analysis are documented in the “Analysis of covariation in gene content” subsection of the Methods with p-value reported in the main text.

Unifrac and the P-test were used to examine beta diversity with p-values reported in Table 2.

Statistical methods for assessing adaptive evolution are described in the “Estimates of dN/dS and tests of adaptive evolution” subsection of the Methods. Degrees of freedom and loglikelihood ratio test statistics that meet a significance criteria of <0.001 using the chi squared distribution are reported in Table 3, Table 4, and Table 5.

(For large datasets, or papers with a very large number of statistical tests, you may upload a single table file with tests, Ns, etc., with reference to sections in the manuscript.)

**Group allocation**

- Indicate how samples were allocated into experimental groups (in the case of clinical studies, please specify allocation to treatment method); if randomization was used, please also state if restricted randomization was applied
- Indicate if masking was used during group allocation, data collection and/or data analysis

Please outline where this information can be found within the submission (e.g., sections or figure legends), or explain why this information doesn’t apply to your submission:

Information on group allocation is detailed in following subsections of the Methods: “Analysis of covariation in gene content” and “Beta diversity analysis”. For gene enrichment analyses we compared genomes with the trait in question to genomes without the trait in question. For beta diversity analyses, we compared genomes from a population in the Pacific Ocean to genomes from populations in the Atlantic Ocean.

Masking was not used during group allocation for the gene enrichment analysis. The beta diversity analysis used random subsampling without replacement as implemented in MOTHUR.

**Additional data files (“source data”)**

- We encourage you to upload relevant additional data files, such as numerical data that are represented as a graph in a figure, or as a summary table
- Where provided, these should be in the most useful format, and they can be uploaded as “Source data” files linked to a main figure or table
- Include model definition files including the full list of parameters used
- Include code used for data analysis (e.g., R, MatLab)
- Avoid stating that data files are “available upon request”

Please indicate the figures or tables for which source data files have been provided:

Figure 1–source data 1. Compressed tar archive (zip format) containing the concatenated codon alignment (fasta format) and tree file (newick format) used to generate Figure 1.

Figure 2–source data 1. Binary matrix containing the raw presence and absence data for each CyCOG in each genome analyzed for Figure 2 and Figure 2–figure supplement 1.

Figure 2–source data 2. Compressed tar archive (zip format) containing input and output files for gene enrichment analysis using BiNGO 3.0.3 (Maere et al. 2005) in Cytoscape 3.4 (Shannon et al. 2003).

Figure 3–source data 1. Compressed tar archive (zip format) containing codon alignments (fasta format) and tree files (newick format) used to generate Figure 3 and Figure 3–figure supplement 1.

Figure 6–source data 1. Compressed tar archive (zip format) containing the alignments (fasta format) and column distance matrices used in the preparation of Figure 6.

Table 1–source data 1. Compressed tar archive (zip format) containing alignments used in CLONALFRAMEML analyses.

Table 2–source data 1. Compressed tar archive (zip format) containing the alignments and group files used in beta diversity analyses.

Table 3–source data 1. Compressed tar archive (zip format) containing example codeml control files, codon alignments (phylip format), and tree files (newick format) used for site model tests of adaptive evolution.

Table 4–source data 1. Compressed tar archive (zip format) containing example codeml control files, codon alignments (phylip format), and tree files (newick format) used for branch-site model tests of adaptive evolution in the HLII clade.

Table 5–source data 1. Compressed tar archive (zip format) containing example codeml control files, codon alignments (phylip format), and tree files (newick format) used for branch-site model tests of adaptive evolution in the LLI clade.
